# Intraoperative transit time flowmetry during off-pump coronary artery bypass grafting: Early outcome of two different anastomosis technique

**DOI:** 10.34172/jcvtr.025.33244

**Published:** 2025-06-28

**Authors:** Abdusalom Abdurakhmanov, Shahboz Buranov, Farkhod Mamataliev, Saidjalol Tursunov, Mustapha Obeid, Ulugbek Ganiev

**Affiliations:** ^1^Republican Research Center of Emergency Medicine, Tashkent, Uzbekistan; ^2^Bukhara Branch of Republican Research Center of Emergency Medicine, Bukhara, Uzbekistan

**Keywords:** Coronary artery bypass grafting, Off-pump coronary artery bypass grafting, Transit time flowmetry

## Abstract

**Introduction:**

Intraoperative transit time flowmetry (TTF) is an essential technique for evaluating graft function during off-pump coronary artery bypass grafting (OPCABG). This study compares graft quality and outcomes using TTF in two surgical approaches: sequential and Y-type saphenous vein grafting.

**Methods:**

A total of 120 patients with triple-vessel coronary artery disease were enrolled and randomized into two groups: Group 1 (n=60) received sequential grafts; Group 2 (n=60) received Y-grafts. Mean graft flow (MGF) and pulsatility index (PI) were measured intraoperatively. Graft function was classified based on flow>15 ml/min and PI<2.5. All patients underwent coronary CT angiography at 48 months to assess graft patency.

**Results:**

Intraoperative TTF showed no significant difference in MGF or PI between graft types, although sequential grafts demonstrated slightly higher flow and lower resistance. Intraoperative graft failure occurred in 1.7% (sequential) vs. 3.3% (Y-grafts). At 48-month follow-up, sequential grafts showed 100% patency, while Y-grafts had a 7.0% occlusion rate. Multivariate analysis identified vessel diameter and conduit type as significant predictors of graft performance; graft configuration (sequential vs. Y) was not independently predictive.

**Conclusion:**

TTF is a valuable intraoperative tool for ensuring graft functionality in OPCABG. While both techniques are viable, sequential grafting demonstrated superior mid-term patency and lower failure rates. These findings support the preferential use of sequential grafting when anatomically feasible and highlight the importance of routine TTF to optimize surgical outcomes.

## Introduction

 Coronary artery bypass grafting (CABG) remains a gold standard in the treatment of coronary artery disease (CAD).^1^ Off-pump coronary artery bypass grafting (OPCABG) is performed without cardiopulmonary bypass (CPB), aiming to reduce associated complications. Early graft failure may occur after coronary artery bypass grafting. Some authors have demonstrated significant variations in patency, and an early graft failure incidence of approximately 5% for internal mammary artery (IMA) grafts, and 11% for vein grafts.^2,3,4^ Intraoperative transit time flowmetry (TTF) provides real-time feedback on graft patency and flow, allowing immediate corrections. Now transit-time flow measurement (TTFM) is the most commonly used method for intraoperative assessment of graft patency during OPCAB. Several studies have reported grafts blood flows by transit-time flowmetry,^5,6^ and TTFM also could predict the graft failure in the short and medium term.^7,8^ Nonetheless, little information is available comparing the surgical results of isolated and sequential grafts. This study compares the intraoperative and mid-term outcomes of sequential and Y-grafts in OPCABG using TTF and computed tomography angiography, focusing on graft failure rates and graft functionality criteria.

## Materials and Methods

###  Patients Selection

 This prospective study included 120 patients who underwent off-pump coronary artery bypass grafting (OPCABG) at our institution. Patients were assigned to two groups based on intraoperative anatomical suitability for the grafting technique: Group 1 (n = 60) received sequential grafts, while Group 2 (n = 60) underwent revascularization using Y-type grafts.

 In Group 1, there were 49 males (82%) and 11 females, with a mean age of 62.1 ± 6.9 years. Group 2 consisted of 45 males (75%) and 15 females, with a mean age of 61.6 ± 8.0 years. Baseline demographic and clinical characteristics were comparable between the two groups.

 Patients were selected according to predefined inclusion and exclusion criteria. Allocation to each group was determined intraoperatively, based on the anatomical characteristics of coronary vessels, including target vessel quality, distribution, and distance between graftable sites, to ensure optimal graft configuration and flow dynamics.

###  Intraoperative Transit Time Flowmetry

 Transit-time flow measurement (TTFM) was used intraoperatively to assess graft function, measuring both the flow rate (ml/min) and pulsatility index (PI) of each graft. The following criteria were applied to interpret graft performance:

- Normal graft function was defined as a flow rate > 15 ml/min and a PI < 2.5.
- Borderline (or questionable) function was defined as a flow between 10–15 ml/min and/or a PI between 2.5–3.5. In such cases, additional hemodynamic testing was performed to evaluate for competitive native coronary blood flow. If flow and PI values improved upon manipulation (e.g., temporary occlusion of native vessels), the graft was considered functionally adequate and left unchanged.
- Graft dysfunction was defined as a flow < 10 ml/min and/or a PI > 3.5. If borderline or abnormal values persisted or worsened despite optimization, the anastomosis was considered dysfunctional and subjected to surgical revision.

This approach allowed for real-time, physiology-based decision-making to ensure optimal graft patency.

###  Follow-up

 All patients were followed up in out-patient clinic at 48 months after surgery. All of them underwent coronary artery CTA to evaluate the grafts.

###  Statistical Analysis

 All statistical analyses were performed using SPSS software, version 16.0 (SPSS Inc., Chicago, IL, USA). Continuous variables were expressed as mean ± standard deviation (SD) for normally distributed data or as median with interquartile range (IQR) for non-normally distributed data, based on results of the Shapiro–Wilk normality test. Categorical variables were presented as frequencies and percentages. For comparison of continuous variables between two related groups, the paired two-tailed *t*-test was applied when data followed a normal distribution; otherwise, the Wilcoxon signed-rank test was used. For categorical variables, comparisons were performed using the chi-square test or Fisher’s exact test as appropriate. A *P* value of less than 0.05 was considered statistically significant. All tests were two-tailed. Effect sizes and 95% confidence intervals (CIs) were reported where relevant to assess the clinical significance of findings.

## Results

 The baseline demographic, clinical, and angiographic parameters were analyzed. A history of previous MI was recorded in 87.7% and 66.3%; three-vessel disease was present in 28(46.7%) and 31(51.7%) patients in Group 1 and Group 2 respectively (Table 1).

**Table 1:** Patient Demographics

**Variable**	**Group 1: Sequential Grafts (n=60)**	**Group 2: Y-Grafts (n=60)**
Age (mean ± SD)	62,1 + 6,9	61,6 ± 8
Gender (M/F)	49/11	45/15
Diabetes (%)	93(44,1%)	111(43,5%)
Hypertension (%)	56 (93,3%)	55 (91,6%)
history of previous MI	53 (87,7%)	40 (66,3%)
Aortic atherosclerosis	37 (61,6%)	39 (65%)
Smoking	44 (73,5%)	42(71,6%)
Chronic kidney disease	10(16,67%)	9 (15,0%)
Triple vessel disease	28(46.7%)	31(51.7)%

###  Intraoperative Transit Time Flowmetry Results

 In all cases intraoperatively we performed TTF, no significant differences in TTFM parameters (MGF, PI) were observed when comparing individual and sequential SV grafting. Intraoperative data listed in Table 2.

**Table 2:** Intraoperative data

**Variable**	**MGF**		**PI**	
	**Sequential grafting**	**Individual Y grafting**	**Sequential grafting**	**Individual Y grafting**
DIAG	51.8 ± 21.0	43.9 ± 15.4	2.5 ± 0.9	1.8 ± 0.7
OM	31.5 ± 21.4	48.8 ± 20.5	2.5 ± 1.2	2.8 ± 1.9
PDA	47.4 ± 21.1	36.5 ± 17.7	2.6 ± 1.7	2.7 ± 1.3
PLA	38.2 ± 15.8	26.3 ± 10.6	2.2 ± 1.2	2.2 ± 0.7
Graft failure	1(1.7%)	2(3.3%)	*P* < 0.05	

DIAG - diagonal artery; OM - obtuse marginal branch of circumflex artery; PDA - posterior descending artery; PLA - left posterior artery; MGF - mean graft flow; PI - pulsatile index.

 Using the recommended method of flowmetric assessment for coronary grafts, dysfunction was identified in 12 anastomoses, resulting in the revision of 12 grafts. The characteristics of the dysfunctional anastomoses are presented in Table 3.

**Table 3:** Anastomotic Dysfunction by Graft Configuration

**Grafted Vessel**	**Y-Graft (n)**	**Sequential (n)**	***P*** **value**
LAD	1	0	0.07
OM	2	2	0.60
RCA	1	1	0.60
PDA	2	3	0.07

LAD – Left anterior descending artery, OM - obtuse marginal branch of circumflex artery, RCA - Right coronary artery, PDA - posterior descending artery. We used Fisher’s exact test. This method was chosen due to the small sample sizes and low expected cell counts (i.e., graft dysfunction events), which violates the assumptions of the chi-square test. The test was applied separately to vessel each category (LAD, OM, RCA, PDA) to determine whether the distribution of dysfunction differed significantly by graft configuration.

 The results of this study demonstrate a lower frequency of graft dysfunction and need for revision in the sequential graft group compared to Y-grafts. Specifically, among the 12 dysfunctional anastomoses identified intraoperatively using TTFM, a disproportionate number occurred in the Y-graft or end-to-side configurations, whereas sequential anastomoses exhibited a notably lower failure rate.

 Sequential anastomoses are typically performed in a side-to-side fashion at a more favorable, oblique angle, which allows for more laminar flow. In contrast, Y-graft configurations may be constructed at sharper or inconsistent angles, predisposing to disturbed flow, increased turbulence, and elevated pulsatility index (PI). This is supported by the higher incidence of elevated PI values observed in Y-grafts during flowmetry.

 Y-grafts are more susceptible to mechanical distortion, especially twisting of the free limb or inadequate alignment with the native vessel. Such mechanical issues were among the primary causes of graft dysfunction during revision surgery. Notably, deformation due to involvement of the coronary artery’s posterior wall in the suture, conduit wall invagination, purse-string effects, and unstable atherosclerotic plaques were frequent findings in dysfunctional Y-graft anastomoses.

 Sequential grafts facilitate continuous distal runoff through multiple anastomoses, creating a siphon effect that enhances mean flow (Qmean) and maintains shear stress. According to flowmetry, sequential grafts demonstrated higher average volumetric flow rates (61.4 ml/min vs. 45.4 ml/min) and lower peripheral resistance (PI: 1.38 vs. 2.45) compared to Y-graft configurations.

 Multivariate regression analysis (Table 4) identified several independent predictors of increased peripheral resistance (PI), a marker of suboptimal graft function. To identify independent predictors of elevated pulsatility index (PI), we performed a multivariate logistic regression analysis. The dependent variable was dichotomized into high PI (≥ 2.5) versus normal PI (< 2.5), based on clinically accepted thresholds for graft function assessment. Independent variables included degree of coronary artery stenosis, graft configuration (sequential vs. Y-type), conduit type (arterial vs. venous), target vessel diameter, and presence of diffuse fibrosis or calcification of the vessel wall. Odds ratios (ORs) with 95% confidence intervals (CIs) were calculated for each variable to determine the strength of association. This model was selected to adjust for potential confounding factors and to evaluate the relative contribution of anatomical and procedural characteristics to increased peripheral resistance:

- Grafted artery diameter had a strong protective effect: every 1.0 mm increase in vessel diameter reduced the likelihood of elevated PI by 79% (OR = 0.21; 95% CI: 0.112–0.395; *P* = 0.001).
- Severe wall fibrosis or calcification was associated with more than double the risk of high PI (OR = 2.25; *P* = 0.05).
- Use of arterial conduits significantly reduced the risk of elevated PI (OR = 0.298; *P* = 0.002).

**Table 4:** Factors Affecting Peripheral Resistance Index (PI)

**Factor**	**OR**	**95% CI**	***P *** **value**
Degree of coronary artery stenosis (%)	0.987	0.975–0.999	0.034
Y-graft with significant stenosis ( > 70%)	0.99	0.84–1.62	0.50
Sequential graft with significant stenosis ( > 70%)	1.02	0.80–1.89	0.50
Conduit type	0.298	0.141–0.632	0.002
Diameter of target artery	0.21	0.112–0.395	0.001
Diffuse fibrosis/calcification of vessel wall	2.25	0.98–5.18	0.05

 Interestingly, neither sequential nor Y-type grafting configurations were significant independent predictors of elevated PI (OR = 1.02 for sequential; *P* = 0.5), suggesting that the observed difference in outcomes is likely related to the anatomical compatibility and technical ease of sequential grafting, rather than the graft design itself. In contrast to PI, multivariate regression analysis of mean flow (Qmean) (Table 5) did not identify significant associations with graft type, conduit type, or the presence of calcification, further emphasizing that PI is a more sensitive marker of graft dysfunction in this context.

**Table 5:** Factors Affecting Mean Flow (Mean)

**Factor**	**OR**	**95% CI**	***P*** ** value**
Degree of coronary artery stenosis (%)	1.001	0.995–1.027	0.195
Y-graft with significant stenosis ( > 70%)	1.06	0.912–1.55	0.18
Sequential graft with significant stenosis ( > 70%)	0.91	0.86–1.065	0.41
Conduit type	0.714	0.314–1.627	0.423
Diameter of target artery	0.463	0.21–1.022	0.05
Fibrosis/calcification of vessel wall	1.21	0.63–2.34	0.56

 To evaluate factors influencing mean graft flow (Qmean), we conducted a multiple linear regression analysis. Qmean (measured in ml/min) was treated as a continuous dependent variable. Independent variables included degree of coronary artery stenosis, graft configuration (sequential vs. Y-type), conduit type (arterial vs. venous), diameter of the target vessel, and the presence of diffuse vessel wall fibrosis or calcification. This model allowed assessment of the relative contribution of each factor to variations in graft flow while controlling for potential confounders. Regression coefficients (β), 95% confidence intervals (CIs), and *P* values were reported. Normality of residuals was assessed, and multicollinearity was evaluated using variance inflation factors (VIFs).

 The lower revision rate in the sequential graft group can be explained by a combination of favorable flow geometry, lower peripheral resistance, reduced mechanical distortion, and more stable anastomotic configurations. Although graft type per se did not emerge as a statistically significant predictor in multivariate models, the cumulative technical and hemodynamic advantages of sequential grafting translated into better clinical performance during intraoperative assessment. These findings reinforce the importance of individualized surgical planning based on coronary anatomy, vessel quality, and conduit characteristics, with preference for sequential grafting when anatomically suitable.

###  Follow up

 To evaluate mid-term outcomes, clinical and imaging follow-up was conducted over a period of up to 48 months postoperatively. Multislice computed tomography (MSCT) angiography was employed to assess graft patency. MSCT angiography was performed in 43 patients from Group 1 (sequential grafting) and 38 patients from Group 2 (Y-type grafting). The detailed findings are presented in Table 6.

**Table 6:** MSCT Angiography Results (Up to 48 Months Postoperative)

**Target Vessel**	**Sequential Grafting**	**Y-Type Grafting**
DIAG	0	1 (2.3%)
OM	0	1 (2.3%)
PDA	0	1 (2.3%)
PLA	0	0

DIAG – Diagonal Artery, OM – Obtuse Marginal Artery, PDA – Posterior Descending Artery, PLA – Posterolateral Artery

 Over the 48-month observation period, occlusion of Y-grafts was detected in 3 patients from Group 2 (7.0%). In contrast, no occlusion of sequential grafts was observed in Group 1. In cases where recurrent angina occurred in Group 1, it was attributed to the progression or occlusion of native coronary arteries or separate linear grafts, rather than the sequential conduits themselves.

 These findings further support the durability and mid-term patency of sequential grafting techniques compared to Y-grafting in off-pump coronary artery bypass surgery.

## Discussion

 Our findings align with previous research highlighting the advantages of sequential grafting in maintaining long-term graft patency. Several studies have demonstrated the superior hemodynamic properties of sequential grafts, which may contribute to their lower failure rates. For example, Joshi et al observed better patency rates with sequential grafts, emphasizing their efficacy in patients with smaller coronary arteries. Similarly, Jingxing Li with coauthors reported superior blood flow in sequential vein grafts compared to individual grafts during off-pump coronary artery bypass, supporting the notion that sequential grafts provide enhanced hemodynamic stability.^8,9^

 The benefits of transit-time flowmetry (TTF) in CABG procedures have been well-documented. Smith et al^7^ reported a significant reduction in graft failure rates when TTF was used, with an overall failure rate of 1.5%. Our study supports these findings, particularly in the sequential graft group, which exhibited a 0% failure rate. Jones et al (2019) also found that TTF use in off-pump coronary artery bypass grafting (OPCABG) reduced postoperative complications, consistent with our observation of no mortality or major adverse cardiac and cerebrovascular events (MACCE) in both groups. The necessity of TTF in preventing graft-related complications has been well-documented. D’Ancona et al demonstrated that TTF is crucial for verifying graft patency intraoperatively, thus preventing immediate and future complications. Kieser et al provided comprehensive evidence supporting the use of TTF in CABG, highlighting its effectiveness in ensuring graft success and reducing the risk of reoperation.^5,6^ Our study adds to this body of evidence by showing that TTF usage in OPCABG leads to better immediate and long-term outcomes, especially with sequential grafts. These results underscore the critical role of TTF in detecting and preventing graft failures, thereby enhancing surgical outcomes.

 Lee JH, et al provided a long-term perspective on graft patency, demonstrating the utility of MSCT angiography in assessing graft patency.^10^ MSCT role in long-term graft patency assessment also reported in some other studies, their data correlates with our findings, indicating that sequential grafts maintain superior patency over extended periods.^1,11^

## Conclusion

 In conclusion, our study underscores the effectiveness of intraoperative transit time flowmetry (TTF) in assessing graft quality and functionality during off-pump coronary artery bypass grafting (OPCABG). The use of TTF provided critical real-time feedback on graft performance, enabling the detection and correction of potential graft failures intraoperatively. Our findings indicate that sequential grafts exhibit superior short-term and long-term patency compared to Y-grafts, with significantly lower intraoperative graft failure rates and better 48-month follow-up outcomes. The data supports the preference for sequential grafting techniques in OPCABG procedures to enhance graft patency and reduce the incidence of complications. Furthermore, the routine implementation of TTF should be considered essential for optimizing surgical outcomes and ensuring the mid-term success of coronary artery bypass grafts.

 Future studies should focus on validating these findings in larger patient populations and through randomized controlled trials to further establish evidence-based guidelines for graft selection and intraoperative assessment protocols in coronary surgery.

## Competing Interests

 The authors declare no competing financial or non-financial interests related to this study.

## Ethical Approval

 This study was approved by the Institutional Ethics Committee of the Republican Research Center of Emergency Medicine, Tashkent, Uzbekistan. All patients provided written informed consent before inclusion in the study, in accordance with the Declaration of Helsinki.
